# Integrative Metabolomic and Transcriptomic Analyses Uncover Metabolic Alterations and Pigment Diversity in *Monascus* in Response to Different Nitrogen Sources

**DOI:** 10.1128/mSystems.00807-21

**Published:** 2021-09-07

**Authors:** Di Huang, Yuhui Wang, Jing Zhang, Huimin Xu, Jing Bai, Huijing Zhang, Xiaolong Jiang, Jian Yuan, Gege Lu, Lingyan Jiang, Xiaoping Liao, Bin Liu, Huanhuan Liu

**Affiliations:** a TEDA School of Biological Sciences and Biotechnology, Nankai University, TEDA, Tianjin, China; b State Key Laboratory of Food Nutrition and Safety, Tianjin University of Science & Technology, Tianjin, China; c Key Laboratory of Food Nutrition and Safety, Tianjin University of Science & Technology, Ministry of Education, Tianjin, China; d Key Laboratory of Systems Microbial Biotechnology, Tianjin Institute of Industrial Biotechnology, Chinese Academy of Sciences, Tianjin, China; Agricultural Biotechnology Research Center

**Keywords:** *Monascus*, nitrogen chemical forms, metabolomics, transcriptomics, pigment diversity, correlation analysis, weighted gene coexpression network analyses

## Abstract

Nitrogen in different chemical forms is critical for metabolic alterations in *Monascus* strains and associated pigment diversity. In this study, we observed that ammonium-form nitrogen was superior in promoting the biosynthesis of *Monascus* pigments (MPs) when compared with nitrate and organic forms. Moreover, with any nitrogen source, the production of yellow and orange pigments was highly synchronized but distantly related to red pigments. However, transcriptional analyses of MP gene clusters suggested a low contribution to MP accumulation, suggesting that MP-limiting factors were located outside the gene cluster. Our metabolomic analyses demonstrated that red pigment biosynthesis was closely related to intracellular amino acids, whereas orange and yellow pigments were associated with nucleotides. In addition, weighted gene coexpression network analyses (WGCNA) based on transcriptomic data showed that multiple primary metabolic pathways were closely related to red pigment production, while several secondary pathways were related to orange pigments, and others were involved with yellow pigment regulation. These findings demonstrate that pigment diversity in *Monascus* is under combined regulation at metabolomic and transcriptomic levels.

**IMPORTANCE** Natural MPs containing a mixture of red, orange, and yellow pigments are widely used as food coloring agents. MP diversity provides foods with versatile colors and health benefits but, in turn, complicate efforts to achieve maximum yield or desirable combination of pigments during the manufacturing process. Apart from the MP biosynthetic gene cluster, interactions between the main biosynthetic pathways and other intracellular genes/metabolites are critical to our understanding of MP differentiation. The integrative multiomics analytical strategy provides a technical platform and new perspectives for the identification of metabolic shunting mechanisms in MP biosynthesis. Equally, our research highlights the influence of intracellular metabolic alterations on MP differentiation, which will facilitate the rational engineering and optimization of MP production in the future.

## INTRODUCTION

The important edible filamentous fungi (1) *Monascus* spp. produce many beneficial secondary metabolites, including a variety of natural *Monascus* azaphilone food colorants and a group of physiologically active substances (monacolins, γ-aminobutyric acid, and dimerumic acid) (2). These molecules are widely used in food and medicines in many Asian countries, such as China, Japan, Korea, and Thailand (1). The well-characterized natural *Monascus* pigments (MPs, molecular structures shown in Fig. S1 in the supplemental material) typically consist of yellow pigments (MYPs, e.g., monascin and ankaflavin), orange pigments (MOPs, e.g., rubropunctatin and monascorubrin), and red pigments (MRPs, e.g., rubropunctamine and monascorubramine). So far, more than 100 pigment structures have been isolated and identified (3, 4).

10.1128/mSystems.00807-21.1 FIG S1 Molecular structures of the six main MPs. Download FIG S1, PDF file, 0.1 MB. Copyright © 2021 Huang et al. 2021 Huang et al. https://creativecommons.org/licenses/by/4.0/ This content is distributed under the terms of the Creative Commons Attribution 4.0 International license.

MPs are broadly applied as colorants in food products, including fermented bean curd, pickles, pastries, sausages, and ham products (2, 5) due to their color diversity, excellent coloring performances, and efficient production from cheap substrates (6). Moreover, MPs also possess a range of beneficial anticancer, antimicrobial, anti-inflammatory, cholesterol-regulating, and antidiabetic activities (7). A recent study confirmed MPs protected against dyslipidemia and nonalcoholic fatty liver disease by regulating components of the liver metabolome and intestinal microbiome (8).

With increasing interest in personalized healthy diets, MP diversity has provided foods with versatile colors and health benefits, permitting the broad development of pigment products with single or specifically combined physicochemical and biological functions. For example, the MYPs monascin and ankaflavin are favored in food applications due to their excellent biological activities, especially for their antioxidative, hypolipidemic, and cholesterol-lowering effects (9). However, the coexistence of different pigments limits the efforts to maximize MYP yields or desirable pigment combinations during manufacturing processes. As of yet, no study has reported the large-scale production of a single pigment. Nevertheless, due to the advantages and economic importance of MPs, considerable efforts have been made in recent decades to elucidate MP biosynthesis mechanisms and examine MP diversity. On one hand, it was reported that several extracellular factors influenced pigment synthesis and diversity (total production and the proportion of each pigment), including fermentation medium (i.e., carbon source [10], glucose stress [11], nitrogen source [12], surfactants [13, 14]), and advanced fermentation processes (dissolved oxygen [15], pH [16], temperature [17], extraction fermentation [18], redox potential [19], light [20], and low-frequency magnetic field treatment [21]). Among them, nitrogen source-based strategies are practical and effective for regulating MP diversity (22–25).

On the other hand, elucidation of MP biosynthetic pathways has provided insights on MP diversity. As previously summarized (26–28), MPs are generally synthesized via polyketide synthesis (PKS), fatty acid synthesis (FAS), and post-synthetic modifications. Diversity in MP molecular genetics arises from the *Monascus* unitary trunk pathway, which guides intermediates toward the classical yellow and orange pigments and features a variety of shunt pathways branching away from the trunk pathway, at highly reactive node compounds. MOPs are initial biosynthetic products which are successively transformed into MRPs by amination or MYPs via redox reactions (6).

However, identifying the dynamic interactions between the main biosynthetic pathway and genes/enzymes/metabolites peripheral to the MP synthetic gene cluster is key to understanding MP differentiation. As a secondary metabolic pathway, MP synthesis competes with other metabolic pathways for a variety of common precursors, enzymes, and energy sources (3), which is the main limiting factor influencing the biosynthesis and accumulation of MPs (4). Therefore, qualitative and quantitative investigations of intracellular gene expression and metabolites are required to understand MP synthesis and diversity.

The omics-based technologies comprising genomics (29, 30), transcriptomics (30–34), and proteomics (34) have been used to identify metabolic mechanisms implicated in carbon starvation stress (30), exposure to blue light (35), gene knockout mutants of *mga* 1–3 (32), and transmembrane secretion of extracellular MPs (34) in *Monascus* strains. These approaches help to elucidate the regulatory mechanisms of MP synthesis and diversity.

To investigate the regulatory mechanisms behind the effects of different nitrogen sources on MP biosynthesis, in this study, we used an integrated metabolomic and transcriptomic strategy, coupled with correlation analyses, to dissect the influence of intracellular metabolic alterations on MP differentiation. This approach provided new insights into the metabolic regulation mechanisms of edible filamentous fungi.

## RESULTS

### Physiological characteristics of the Monascus purpureus M7 strain in response to various nitrogen chemical forms.

**FIG 1:**
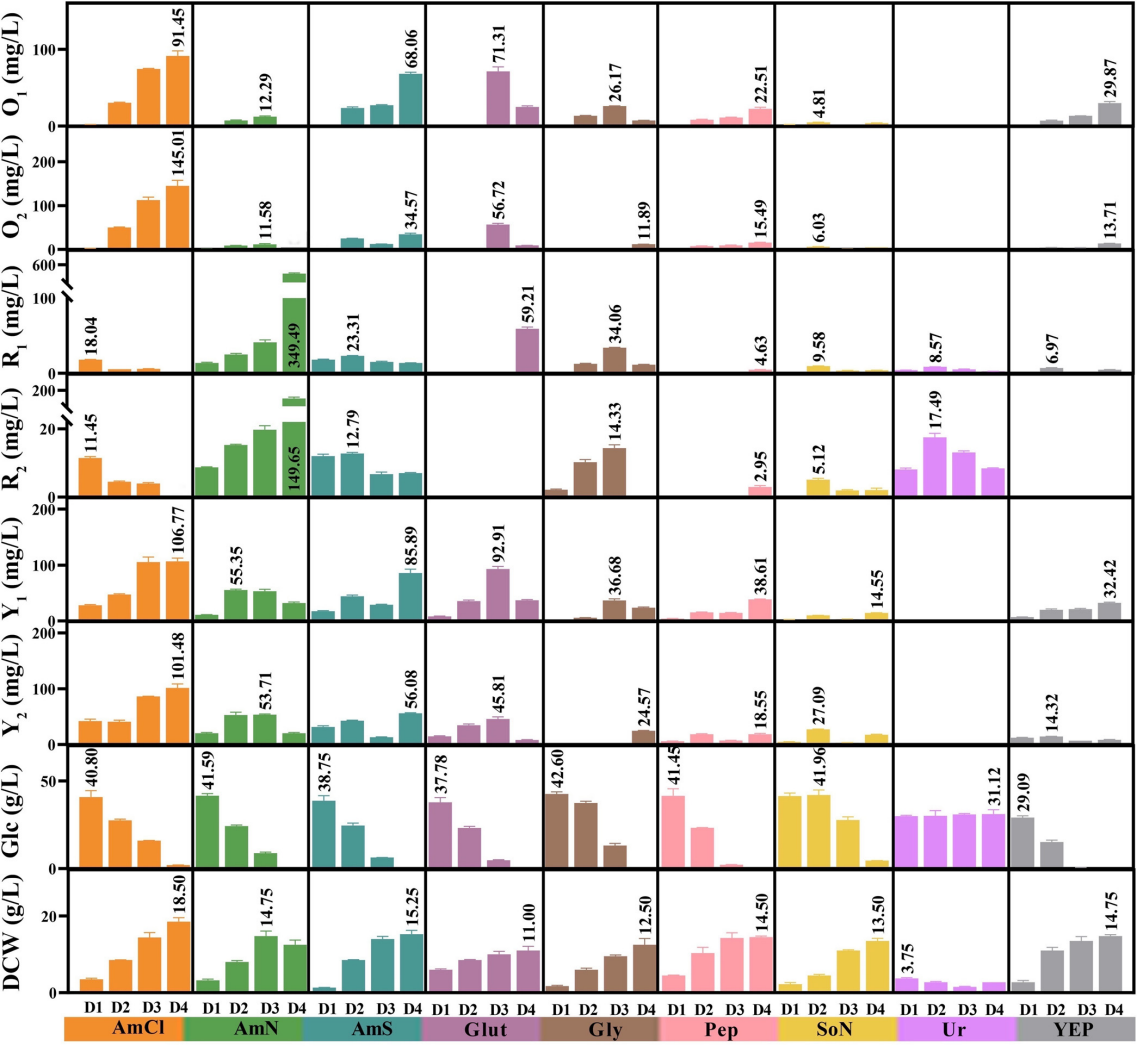
Physiological characteristics of the *M. purpureus* M7 strain in response to nine different nitrogen sources in various chemical forms. D1, D2, D3, and D4 denote samples at days 1 to 4, respectively. RG, residual glucose; Y_1_, monascin; Y_2_, ankaflavin; O_1_, rubropunctatin; O_2_, monascorubrin; R_1_, rubropunctamine; R_2_, monascorubramine. Error bars represent the standard deviation of the mean (*n *= 3 independent experiments). Results of Student's *t* test between two groups are supplied in Table S2 in the supplemental material.

Different nitrogen forms were shown to contribute to wide variations in strain physiological characteristics (Fig. 1). Urea (Ur) was not a suitable nitrogen source, as it depressed biomass and glucose absorption, and only a small amount of MRPs (R_1_ and R_2_) was detected. Sodium nitrate (SoN) was utilized by *Monascus*, but most production levels of the six MPs (R_1_, R_2_, Y_1_, Y_2_, O_1_, and O_2_) were lower than other nitrogen sources (except Ur). When the three ammonium salts, ammonium sulfate (AmS), ammonium chloride (AmCl), and ammonium nitrate (AmN) were used, MP production was higher than other sources. In particular, R_1_ and R_2_ levels were 349.5 mg/liter and 149.7 mg/liter, respectively, in response to AmN nitrogen input. The largest biomass production (18.5 g/liter), Y_1_ (106.8 mg/liter), Y_2_ (101.5 mg/liter), O_1_ (91.5 mg/liter), and O_2_ (145.0 mg/liter) occurred for AmCl. In comparison, the two amino acids, glycine (Gly) and glutamate (Glut) and two protein hydrolysates, peptone (Pep) and yeast extract powder (YEP), resulted in markedly lower MP production than ammonium salts. These findings suggested significant physiological characteristics are highly varied across chemical forms of nitrogen inputs.

**FIG 2:**
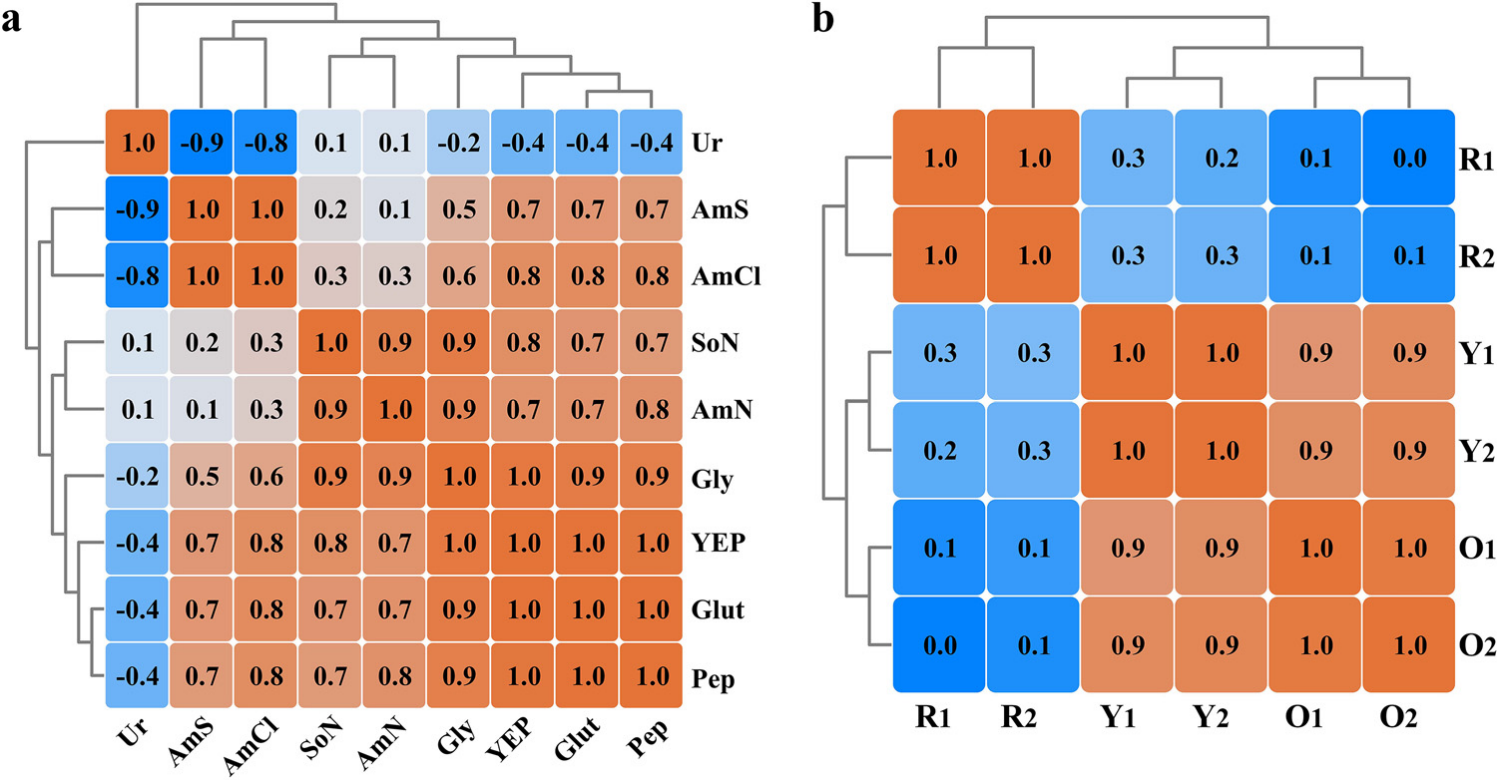
Pearson correlation analyses of physiological *M. purpureus* M7 characteristics in response to nine different nitrogen sources in various chemical forms. (a) Cluster dendrogram of nitrogen sources with different pigment production profiles. (b) Cluster dendrogram of MP production using different nitrogen sources. The blue/orange colors refer to Pearson correlation coefficients with a range of −1.0 to 1.0.

To visualize associations between nitrogen sources and MP production, Pearson correlation analyses were conducted based on MP production over a 1- to 4-day fermentation period (Fig. 2a). The phenotypic changes from different nitrogen sources were clustered into four groups, with a threshold correlation coefficient *r *> 0.8 (*P < *0.05), i.e., Glut-Pep-YEP-Gly, AmCl-AmS, Gly-SoN-AmN, and Ur. High correlations within each group suggested similarities in MP production profiles.

Additionally, by calculating Pearson correlation coefficients with a random four or more samples (Table S1), correlations between MYPs and MOPs were evaluated at the high level (most *r *> 0.8, Fig. 2b), whereas coefficients between MYP/MOP and MRP were near 0, indicating MOP and MYP were strongly correlated, while both were weakly correlated with MRP. This finding mathematically supported the occurrence of an intertransformational relationship between MOP and MYP, as proposed by Xiong et al. (14), and a biosynthetic mechanism of MPs described by Chen et al. (6).

10.1128/mSystems.00807-21.1 TABLE S1 Calculation of correlation coefficients between the MP productions across different samples by MATLAB programming. Download Table S1, XLSX file, 0.01 MB. Copyright © 2021 Huang et al. 2021 Huang et al. https://creativecommons.org/licenses/by/4.0/ This content is distributed under the terms of the Creative Commons Attribution 4.0 International license.

10.1128/mSystems.00807-21.1 TABLE S2 Student′s t test for independent samples of phenotypic characteristics. Download Table S2, XLSX file, 0.03 MB. Copyright © 2021 Huang et al. 2021 Huang et al. https://creativecommons.org/licenses/by/4.0/ This content is distributed under the terms of the Creative Commons Attribution 4.0 International license.

Collectively, although MP biosynthesis varied in response to different nitrogen sources, it was still classifiable by MP production profile or nitrogen source, suggesting an underlying mechanism for MP synthesis, especially for MOP and MYP production, as they displayed a positive linear co-occurrence. Thus, to probe relationships between nitrogen sources and MP biosynthesis, integrated metabolomic and transcriptomic analyses coupled with correlation analyses were performed to ascertain MP mechanisms and associated relationships with intracellular metabolic alterations induced by different nitrogen sources.

### Overview of metabolomic and transcriptomic analyses.

**FIG 3:**
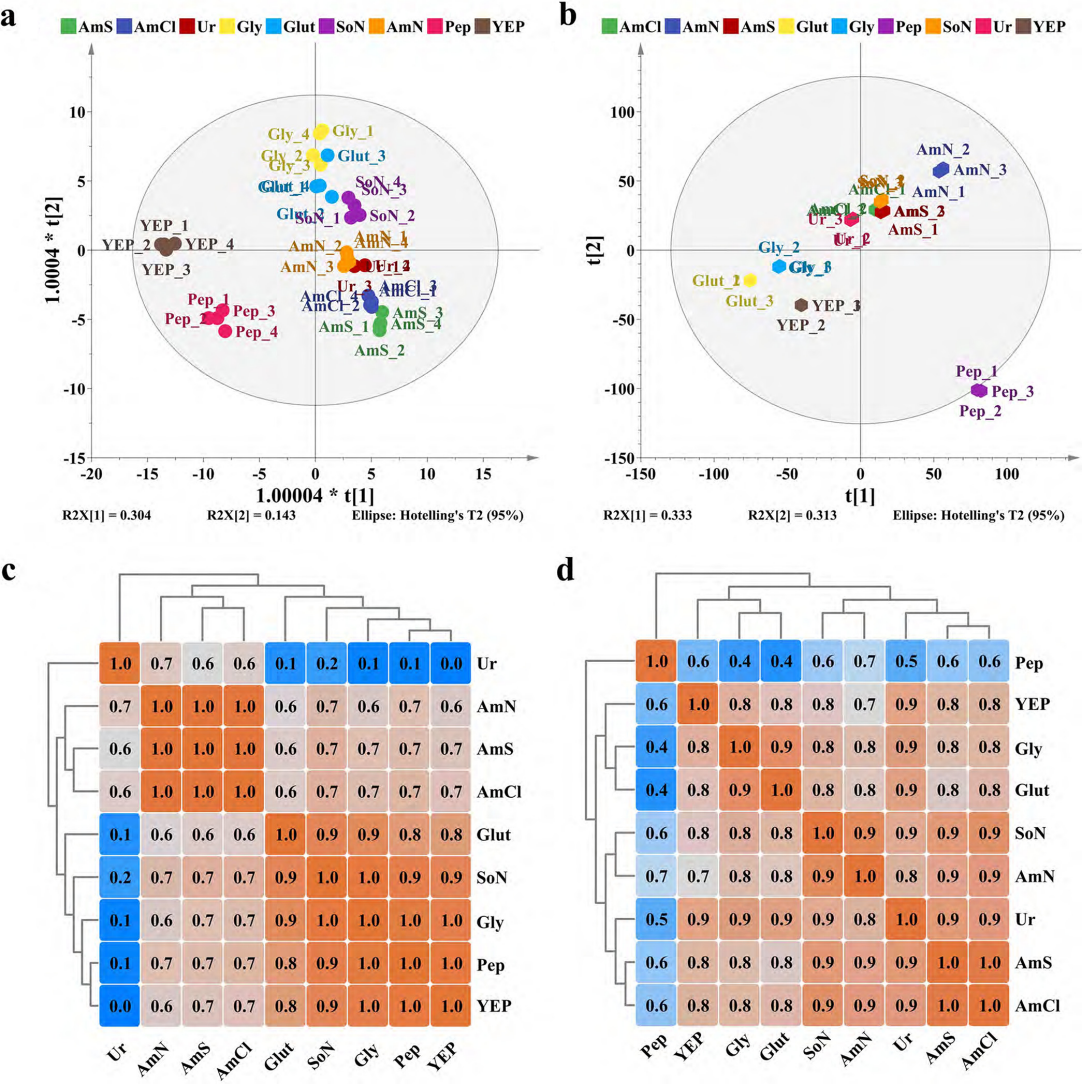
Overview of metabolomic and transcriptomic profiles in response to nitrogen sources in various chemical forms. (a) orthogonal partial least-squares discriminant analysis (O_2_PLS-DA) based on metabolomic data. (b) O_2_PLS-DA based on transcriptomic data. (c) Cluster dendrogram of nitrogen source conditions with transcriptome profiles assessed by Pearson correlation analysis. (d) Cluster dendrogram of nitrogen source conditions with metabolome profiles assessed by Pearson correlation analysis. The blue/orange colors refer to Pearson correlation coefficients with a range of −1.0 to 1.0.

Metabolomic and transcriptomic samples were harvested on the 2nd day of fermentation. Metabolomic samples were analyzed by ultra-high-performance liquid chromatography coupled to a mass spectrometer (UHPLC-MS), which identified 130 metabolites, including organic acids, phosphoric acid compounds, amino acids, nucleotides, and corresponding derivatives (Table S3). As for transcriptomic analyses using the Illumina Hiseq platform, 7,490 genes were mapped to the reference genome (Table S4). An orthogonal partial least-squares discriminant analysis (O_2_PLS-DA) was performed to classify high-dimensional omics data (Fig. 3a, metabolomic data: fit index *R*^2^ = 0.833; predictivity index *Q*^2^ = 0.680; *n* = 6, and Fig. 3b, transcriptomic data: *R*^2^ = 0.942; *Q*^2^ = 0.846; *n* = 6). Metabolomic/transcriptomic samples in each group were clustered, indicating they were well repeated and suitable for further analysis.

10.1128/mSystems.00807-21.1 TABLE S3 Metabolomic data. Download Table S3, XLSX file, 0.1 MB. Copyright © 2021 Huang et al. 2021 Huang et al. https://creativecommons.org/licenses/by/4.0/ This content is distributed under the terms of the Creative Commons Attribution 4.0 International license.

10.1128/mSystems.00807-21.1 TABLE S4 Transcriptomic data. Download Table S4, XLSX file, 2 MB. Copyright © 2021 Huang et al. 2021 Huang et al. https://creativecommons.org/licenses/by/4.0/ This content is distributed under the terms of the Creative Commons Attribution 4.0 International license.

Then, Pearson correlation coefficients were calculated to analyze intracellular metabolomic and transcriptomic alterations in response to nitrogen sources (Fig. 3c and d). The metabolomes of AmS, AmN, and AmCl were tightly clustered (*r *> 0.99), while Glut, Gly, SoN, Pep, and YEP were clustered into another group (*r *> 0.8). In line with its physiological characteristics (Fig. 1), Ur was distinct from all clusters at the metabolomic level (Fig. 3c).

The correlation coefficients of transcriptomic profiles under any two nitrogen sources (Fig. 3d) were >0.7 (*P < *0.05), except for Pep. This demonstrated a relatively steady metabolism at the gene expression level, even though *Monascus* was exposed to different nitrogen inputs. However, these data implied that extracellular phenotypic changes, such as MP production, were putatively dominated by the intracellular differential expression of a small number of genes, even free from direct regulation of gene expression.

To further decipher the synthesis and regulatory mechanisms of pigment diversity, transcriptional profiling of the MP biosynthetic gene cluster was prioritized for in-depth analysis, given that gene cluster expression was a prerequisite for MP production.

### Correlation analyses between gene cluster expression and MP production.

**FIG 4:**
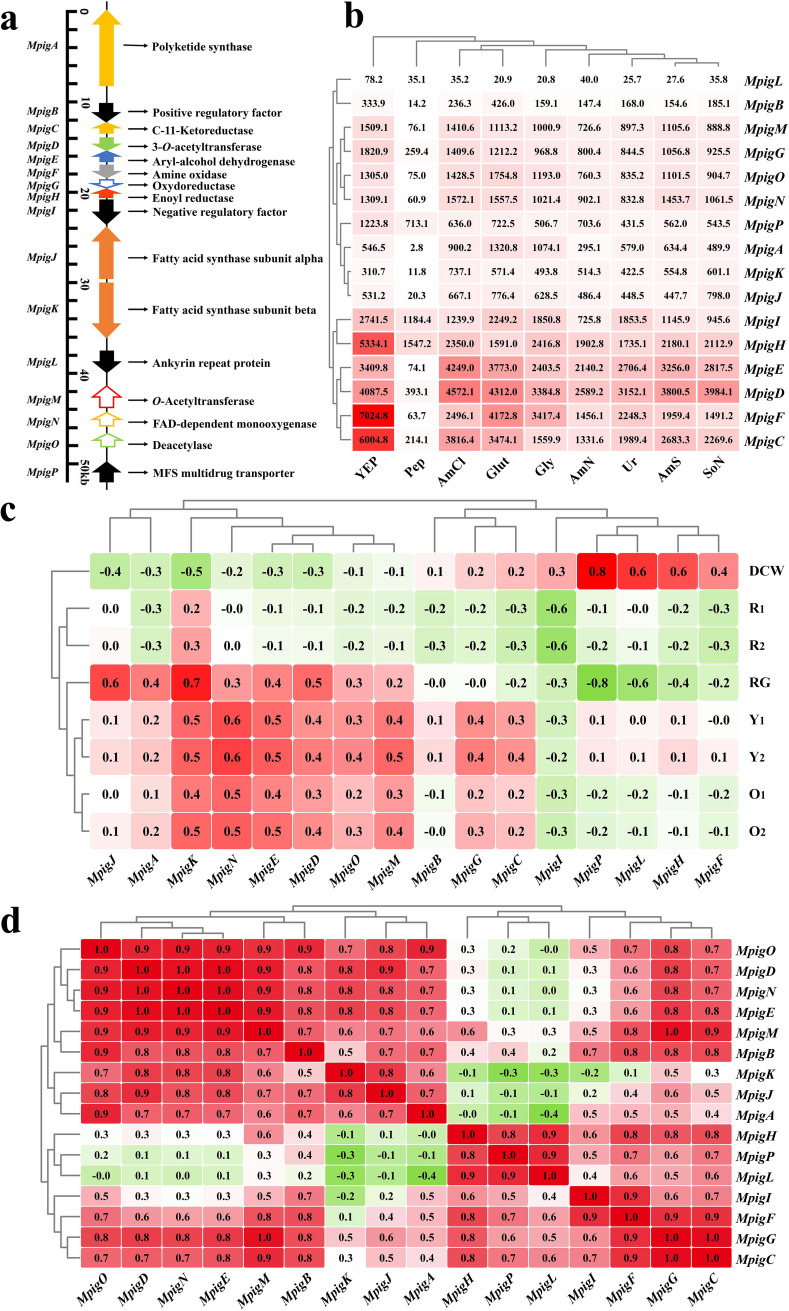
Transcriptional analysis of the MP synthetic gene cluster. (a) Gene cluster structure and functional annotation. (b) Expression profiles (FPKMs). The color density is proportional to the FPKM value. (c) Pearson correlation analysis of gene expression and phenotypic characteristics, including MP production, dry cell weight, and residual glucose. (d) Coexpression analysis in the MP gene cluster. The green/red colors in Fig. 4c and d refer to Pearson correlation coefficients with a range of −1.0 to 1.0.

The MP synthesis gene cluster contains 16 highly conserved genes, where *MpigB*, *I*, *L*, and *P* encode transportation proteins and regulatory factors, and the remaining genes are structural, encoding enzymes involved in MP synthesis and postsynthetic modifications (Fig. 4a). Unexpectedly, we observed no significant positive correlations between overall expression of the gene cluster and MP production (Fig. 4b). For example, when Pep and AmN were used as nitrogen sources, most genes were significantly downregulated despite MP production levels higher than Ur (Fig. 1). For YEP, *MpigH*, *F*, *C*, *I*, *L*, *G*, and *P* were strongly upregulated when compared with other nitrogen sources, but MP production was relatively low. In addition, the highest MP production levels were observed for AmS and AmCl, but their gene cluster expression levels were considerably different. Clearly, the overall expression of the gene cluster displayed weak relationships with pigment production, suggesting limited critical genes may be worthy of attention.

To identify these genes, correlation analyses were performed on gene cluster expression and phenotypic characteristics (Fig. 4c). For MYP and MOP, Y_1_, Y_2_, O_1_, and O_2_ had a closer relationship with gene cluster expression than the MRPs, R_1_ and R_2_ because a number of structural genes (*MpigK*, *-N*, *-E*, *-D*, *-O*, *-M*, *-G*, and *-C*) were positively correlated with both MYP and MOP production. Of these, *MpigK* (encoding fatty acid synthase subunit β involved in pigment side chain synthesis) and *MpigN* (encoding FAD-dependent monooxygenase, involved in oxidation of the polyketide chromophore) showed the closest correlation with MYPs and MOPs (*r *= 0.5 to 0.6). Moreover, *MpigI*, a known negative regulatory factor (6), was negatively related to R_1_ and R_2_ (*r* < −0.6, *P < *0.05) and also Y_1_, Y_2_, O_1_, and O_2_, but with weaker associations (*r =* −0.3 to −0.2).

Due to the fact that the coordination of genes within the cluster provides the necessary genetic basis for the synthesis and differentiation of pigment molecules, and that a group of genes coexpressed across diverse experimental conditions may have similar roles during cellular activity, we performed a coexpression analysis in the gene cluster. Hierarchical clustering (Fig. 4d) revealed that genes were distinctly divided into two subsets, with *MpigH* at the boundary, i.e., *MpigA-B-D-E-J-K-M-N-O* and *MpigH-P-L-I-F-G-C*. The latter gene set included the regulatory genes *MpigP, -L*, and *-I* and the dehydrogenase genes *MpigH*, *-F*, *-G*, and *-C*, of which *MpigH* and *-F* were critical for MOP and MYP differentiation. The versatile precursor from the major MP pathway can be converted into MOPs by reduction with MpigF or into MYPs by oxidation with MpigH. The former gene set mainly included structural genes that were responsible for the synthesis of the polyketide core, the fatty acid side chain, and the postsynthetic modification of intermediates. These highly coexpressed genes with clearly distinct biological functions suggested a directional regulation of MP production by the gene cluster, although pigment synthesis, especially the MRPs (R_1_ and R_2_), was not highly correlated with gene cluster expression. Nevertheless, expression of the MP biosynthetic gene cluster was an essential factor for MP accumulation and suggested key factors limiting MP synthesis may be outside the gene cluster. Therefore, a further in-depth analysis was conducted.

### Correlation analyses between central carbon metabolism and MP production.

**FIG 5:**
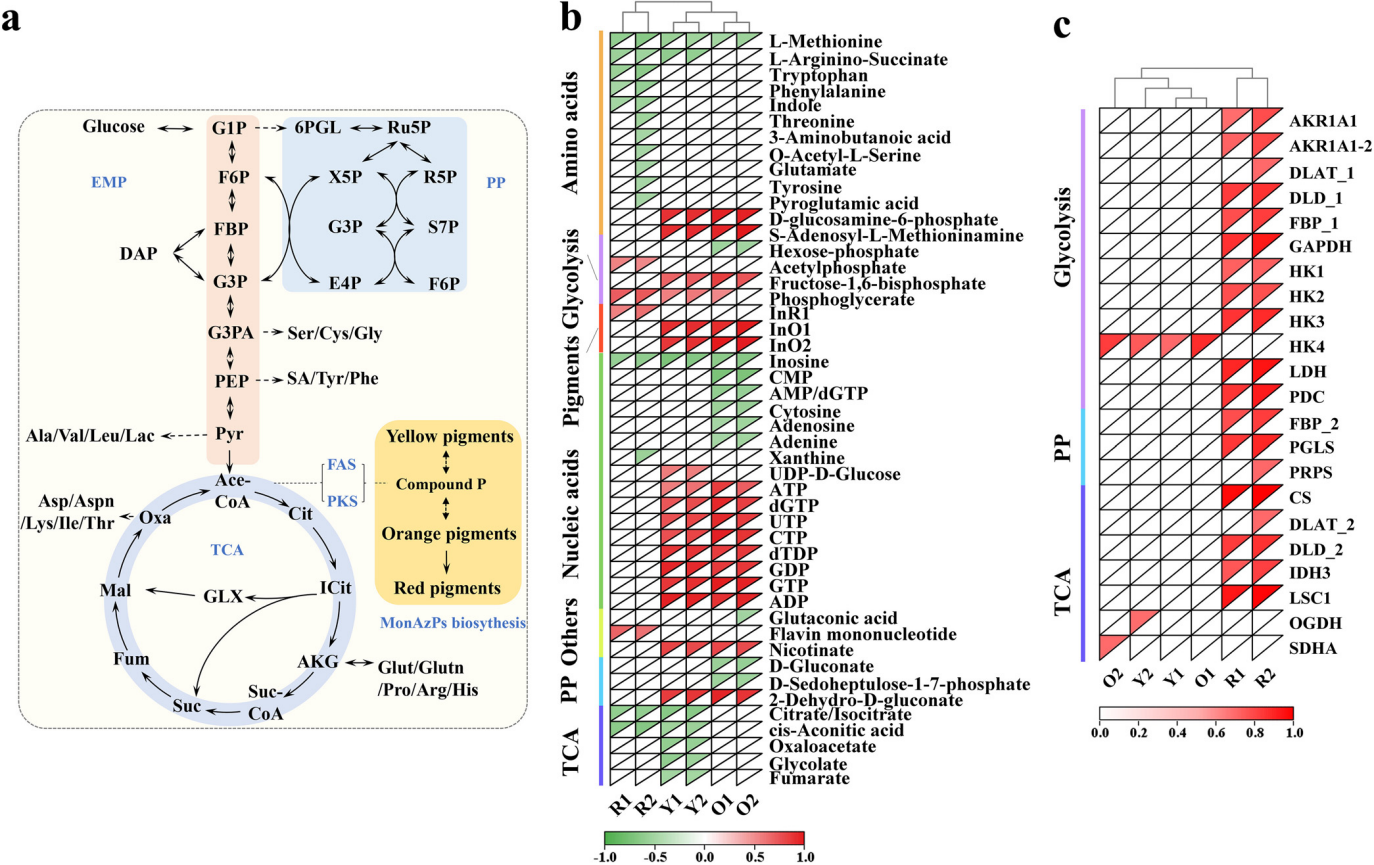
Analysis of relationships between MP biosynthesis and metabolites/gene expression in central carbon metabolism. (a) Central carbon metabolism and related amino acids. (b) Pearson correlations between intracellular metabolites and phenotypes (MP production). Metabolites with correlation coefficients *|r|* > 0.6 are shown. AA, amino acid; EMP, Embden Meyerhof pathway; NT, nucleotides; PP, pentose phosphate pathway; TCA, tricarboxylic acid cycle; OT, others. (c) Correlations between 66 gene expression profiles and phenotypes involving EMP, PP, and TCA pathways, where genes with *|r|*>0.6 are shown. G1P, glucose 1-phosphate; 6PGL, gluconolactone 6-phosphate; Ru5P, ribulose 5-phosphate; R5P, ribose 5-phosphate; X5P, xylose 5-phosphate; F6P, fructose 6-phosphate; FBP, fructose 1,6-diphosphate; DAP, dihydroxyacetone phosphate; G3P, glyceraldehyde 3-phosphate; G3PA, glycerate 3-phosphate; PEP, phosphoenolpyruvate; Pyr, pyruvate; S7P, sedoheptulose 7-phosphate; E4P, erythrose 4-phosphate; Ser, serine; Cys, cysteine; Gly, glycine; SA, shikimic acid; Tyr, tyrosine; Phe, phenylalanine; Ala, alanine; Val, valine; Leu, leucine; Lac, lactate; Asp, aspartate; Aspn, asparagine; Lys, lysine; Ile, isoleucine; Thr, threonine; Cit, citrate; ICit, isocitrate; AKG, α-ketoglutarate; Suc, succinate; Fum, fumarate; Mal, malate; Oxa, oxaloacetic acid; GLX, gloxylate,; Glu, glutamate; Glutn, glutamine; Pro, proline; Arg, arginine; His, histidine; tktA, transketolase; ACLY, ATP citrate (pro-S)-lyase; AKR1A1, alcohol dehydrogenase (NADP^+^); CS, citrate synthase; DLAT, pyruvate dehydrogenase E2 component; DLD, dihydrolipoamide dehydrogenase; FBP, fructose-1,6-bisphosphatase I; GAPDH, glyceraldehyde 3-phosphate dehydrogenase; HK, hexokinase; IDH1, isocitrate dehydrogenase; IDH3, isocitrate dehydrogenase (NAD^+^); LDH, L-lactate dehydrogenase; LSC1, succinyl-CoA synthetase alpha subunit; OGDH, 2-oxoglutarate dehydrogenase E1 component; pckA, phosphoenolpyruvate carboxykinase (ATP); PDC, pyruvate decarboxylase; PGD, 6-phosphogluconate dehydrogenase; PGLS, 6-phosphogluconolactonase; pgm, phosphoglucomutase; PK, pyruvate kinase; PRPS, ribose-phosphate pyrophosphokinase; SDHA, succinate dehydrogenase (ubiquinone) flavoprotein subunit; TPI, triosephosphate isomerase (TIM); xfp, xylulose-5-phosphate/fructose-6-phosphate phosphoketolase. The green/red colors refer to Pearson correlation coefficients with a range of −1.0 to 1.0.

Central carbon metabolism (Fig. 5a) is the core metabolic module providing carbon skeletons, energy for cell growth and metabolism, and precursors (i.e., acetyl-CoA, malonyl-CoA, and amino acids) for MP synthesis. Here, correlation analyses of metabolite abundance and pigment production were performed, with metabolites at *|r|* < 0.6 filtered out. In total, 47 metabolites were identified with a significant relationship to MP production (Fig. 5b). In addition, 66 genes were mapped to the Embden-Meyerhof pathway (EMP), the pentose phosphate (PP) pathway, and the tricarboxylic acid (TCA) cycle. After filtering, 22 genes (*|r|* > 0.6) were correlated with MP production, including 12 genes in the EMP pathway, 7 in the TCA cycle, and 3 in the PP pathway (Fig. 5c). The two MRPs, R_1_ and R_2_, were more closely related to central carbon metabolism than MOPs or MYPs at the transcriptomic level. At the metabolomic level, a number of amino acids and their derivatives (l-methionine, l-arginino-succinate, tryptophan, phenylalanine, indole, 3-aminobutanoic acid, *O*-acetyl-l-serine, glutamate, pyroglutamic acid, threonine, and tyrosine) had negative effects on MRP biosynthesis (especially R_2_; Fig. 5b), whereas a number of nucleotides (UDP-d-Glucose, ATP, dGTP, UTP, CTP, dTDP, GDP, GTP, and ADP) were positively associated with MYP and MOP biosynthesis. These findings demonstrated that the production of MRPs was related to amino acids at the metabolomic level and central carbon metabolism gene expression at the transcriptomic level, whereas the other MPs were regulated by nucleotides and were almost free from central carbon metabolism influences.

### Weighted gene coexpression network analysis (WGCNA) identified correlations between gene transcription and MP biosynthesis.

Targeted analysis of MP gene cluster expression and central carbon metabolism indicated that the production of each MP was regulated in its own unique way. In addition to pathways directly related to pigment synthesis, other sophisticated regulation strategies by *Monascus* were extracted from genome-scale transcriptional data, potentially supplying more genetic mechanisms on pigment synthesis and diversity. Therefore, a WGCNA was implemented.

### Construction of WGCNA.

**FIG 6:**
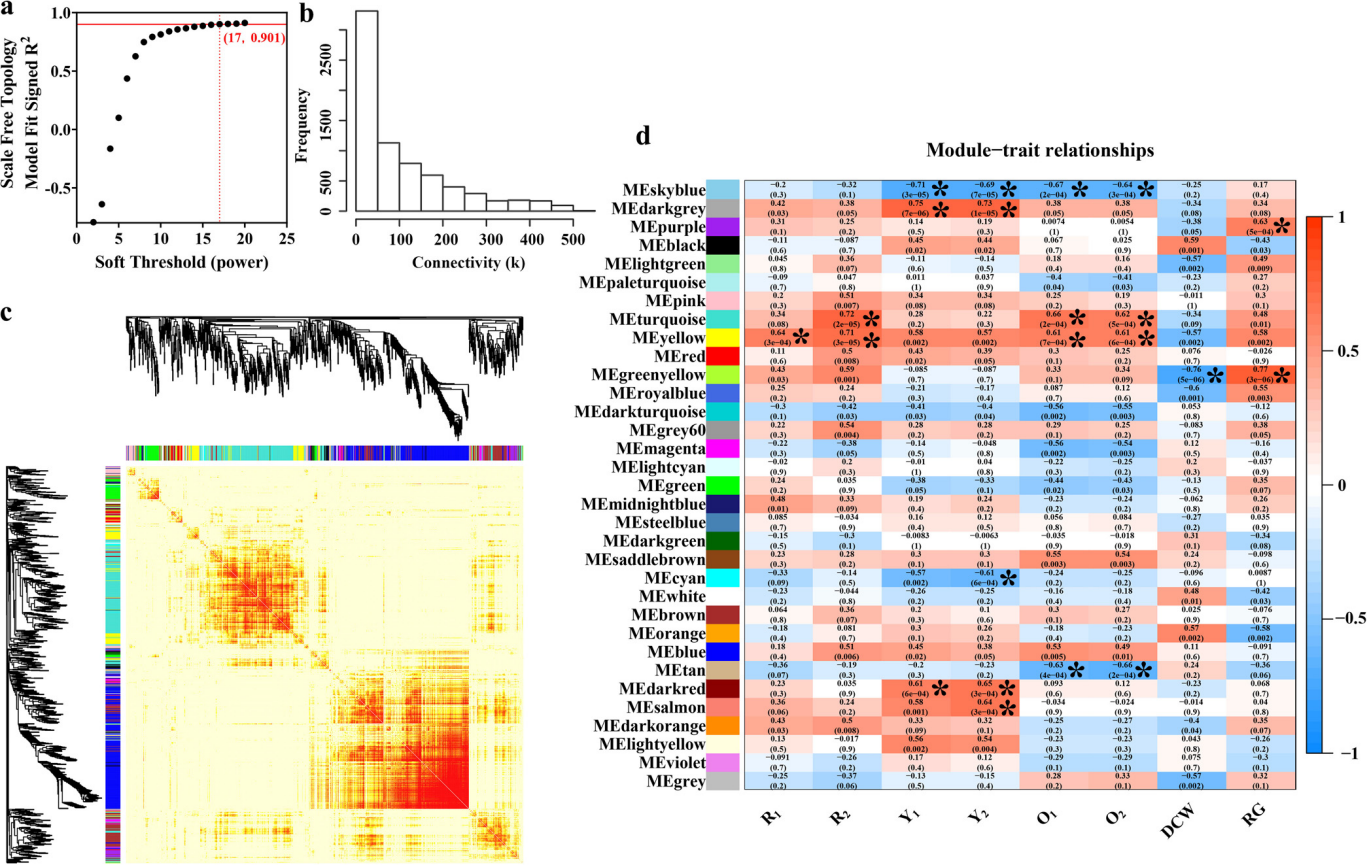
Construction of the WGCNA model. (a) Soft threshold (power) versus scale-free topology model fit significance. (b) Frequency distribution of connectivity (k). (c) Network heatmap. One thousand genes were selected and displayed randomly. (d) Module-trait associations. Each row corresponds to a module eigengene and each column to a trait. The correlation coefficient *r* and *P* values were calculated using module-trait relationships in the heatmap from Fig. 6c.

WGCNA is a systematic biological method permitting the identification of gene association-patterns among different samples, the determination of highly coexpressed gene sets, and identification of candidate biomarker genes, according to associations between genes and phenotypes (36, 37). For WGCNA construction, the soft threshold was set to 17 for the criterion of free-scale topology (Fig. 6a and b), a phenomenon observed in gene expression networks and a variety of complex biological systems, where the distribution of gene relationships follows a power decay law, i.e., genes with the highest number of connections occur least frequently (38).

A cluster dendrogram of partially expressed genes was generated (Fig. 6c). Based on hierarchical clustering, dynamic branch cutting methods, and the Topological Overlap Matrix (TOM), 7,490 genes were grouped into 33 modules. Module-trait relationships were analyzed using correlations between the module eigengenes and eight physiological characteristics (MP production, DCW, and RG), which identified coexpressed gene modules with significant correlations to physiological characteristics. The WGCNA modules with correlation coefficients |*r*| ≥ 0.60 and *P ≤ *0.001 were highly associated with physiological characteristics and resulted in 22 module-trait relationships (asterisks in Fig. 6d, Table S5). The generated modules overlapped with respect to closely related traits, such as O_1_/O_2_ and Y_1_/Y_2_.

To validate the transcriptomic result, several representative genes of different WGCNA modules were selected and subject to quantitative reverse transcription-PCR (qRT-PCR) analysis. Gene annotation, PCR primers, qRT-PCR results, and fragments per kilobase of transcript sequence per million base pairs sequenced (FPKM) from transcriptomic analysis are supplied in Table S6. Pearson correlation analysis was conducted to check the consistency of gene expression between qRT-PCR and transcriptome sequencing (RNA-seq). As the result, gene expression detected by qRT-PCR was highly correlated with that by RNA-seq for each gene (correlation coefficients greater than 0.75). It demonstrated the reliability of transcriptomic data.

10.1128/mSystems.00807-21.1 TABLE S5 Gene-trait relationships identified by WGCNA. Download Table S5, XLSX file, 0.2 MB. Copyright © 2021 Huang et al. 2021 Huang et al. https://creativecommons.org/licenses/by/4.0/ This content is distributed under the terms of the Creative Commons Attribution 4.0 International license.

10.1128/mSystems.00807-21.1 TABLE S6 Validation of the transcriptomic analysis. Download Table S6, XLSX file, 0.01 MB. Copyright © 2021 Huang et al. 2021 Huang et al. https://creativecommons.org/licenses/by/4.0/ This content is distributed under the terms of the Creative Commons Attribution 4.0 International license.

### Pathway enrichment based on WGCNA modules.

**FIG 7:**
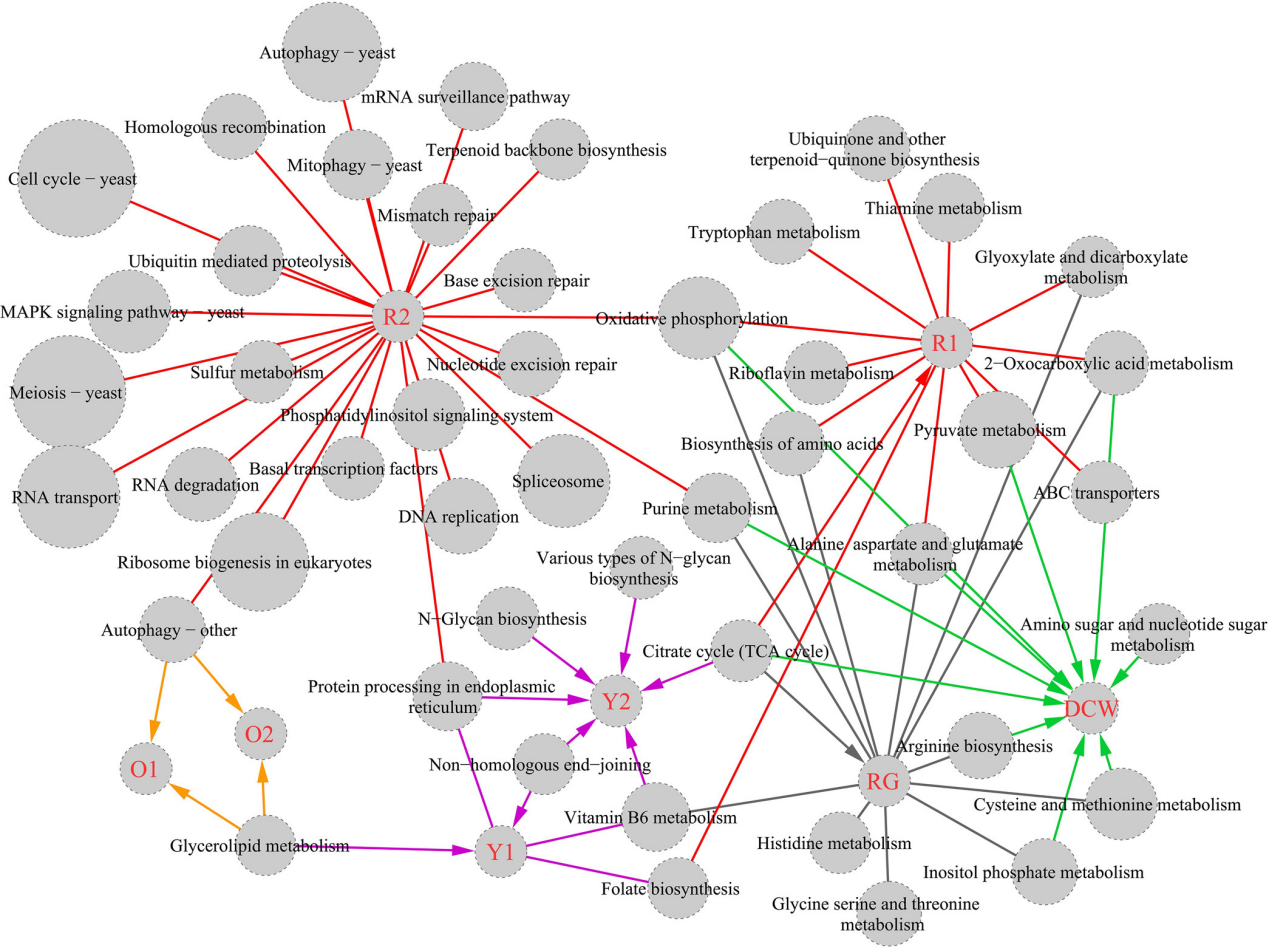
KEGG pathway enrichment based on trait-related gene modules in WGCNA. Node size represents the −log(*p*) value of each pathway item (*P < *0.05). Associations between trait-related gene modules in WGCNA and eight traits (MP production, DCW, and RG) were provided with biological significance, described by KEGG pathways, and linked with red/gray edges representing positive/negative correlations.

To dissect the biological significance of gene modules identified by WGCNA, modules with highly positive correlations to each trait were combined and subjected to Kyoto Encyclopedia of Genes and Genomes (KEGG) pathway enrichment, as were negatively correlated gene modules (Table S7). Thus, genes positively correlated with RG were mainly involved with primary metabolism, i.e., alanine, aspartate, and glutamate metabolism, biosynthesis of amino acids, cysteine, and methionine metabolism, the TCA cycle, and glyoxylate and dicarboxylate metabolism (Fig. 7). KEGG pathways related to dry cell weight (DCW) included purine metabolism, arginine biosynthesis, carbon metabolism, TCA cycle, alanine, aspartate, and glutamate metabolism. Also, most overlapped with RG.

10.1128/mSystems.00807-21.1 TABLE S7 KEGG pathway enrichment. Download Table S7, XLSX file, 0.01 MB. Copyright © 2021 Huang et al. 2021 Huang et al. https://creativecommons.org/licenses/by/4.0/ This content is distributed under the terms of the Creative Commons Attribution 4.0 International license.

For O_1_ and O_2_, gene modules were enriched for autophagy and glycolipid metabolism. Autophagy in eukaryotes facilitates the turnover of intracellular materials, where damaged proteins or organelles are enveloped in autophagic double membrane vesicles and transported to vacuoles for degradation and recycling (39). Glycolipid metabolism, especially glycolipid degradation, could channel carbon flux from triacylglycerols into polyketides (40), which are important precursors for MPs. Both pathways were classified as secondary metabolism pathways.

For R_1_, gene sets were enriched with primary metabolic pathways, such as oxidative phosphorylation, pyruvate metabolism, the TCA cycle, and thiamine metabolism. Of these, oxidative phosphorylation displayed the highest significance (*P = *10^−10.6^). Basal cellular processes such as the cell cycle, meiosis, RNA transport, and ribosome biogenesis, which controlled *Monascus* growth and development, were related to R_2_ production. These findings confirmed that MRPs were highly associated with central carbon metabolism.

In contrast with O_1_/O_2_ and R_1_/R_2_, Y_1_/Y_2_-related gene modules were involved with vitamin B6 metabolism, *N*-glycan biosynthesis, and nonhomologous end joining. Vitamin B6 and *N*-glycan biosynthesis are involved in amino acid metabolism and *N*-linked glycosylation during protein posttranslational modification, respectively. Nonhomologous end joining is necessary for the repair of DNA double-strand breaks against genotoxic agents. Although these metabolic pathways were not directly involved in cell structure, they were essential for normal physiological and biochemical activities.

## DISCUSSION

Nitrogen sources supply basic materials for the synthesis of biological macromolecules, including proteins and nucleic acids, and other nitrogen-containing compounds. These factors are crucial for *Monascus* spp. growth and development and promote the synthesis of pigments, especially MRPs, by nonenzymatic ammoniation reactions (6). Collectively, nitrogen sources exert regulatory effects on pigment synthesis when combined with other factors such as culture conditions, *Monascus* strains, and medium composition. For example, Patrovsky et al. (41) reported that when using peptone as a nitrogen source with an initial pH of 2.5, total MYP/MOP production by *M. purpureus* was much higher than ammonium sulfate or sodium nitrate. In a study conducted by Shi et al. (42), at the initial pH 6.5, ammonium sulfate caused Monascus anka to synthesize major MRPs, accompanied by small amount of MYPs, but when peptone was used, the major components were MYPs along with a few MRPs. Neither peptone nor ammonium sulfate promoted MOP production by Monascus ruber, whereas orange-red pigments were observed using both sources, with higher yields from peptone. However, in this study, the ammonium salt nitrogen sources AmS and AmCl strongly promoted MYP and MOP production, whereas AmN supported MRP production. Pep, YEP, Gly, Glut, SoN, and Ur were considerably less effective. It was clear in this study that ammonium ions were better than organic nitrogen sources for *Monascus* growth and MP production.

In general, the interactions of multiple factors have made *Monascus* MP investigation considerably more complex. Fortunately, a clear goal, i.e., the yield and ratio of pigments, is being pursued, which allows the dissection of the deep mechanism for pigment synthesis and regulation from the level of transcriptome and metabolome. Undoubtedly, each nitrogen source triggers a different series of intracellular metabolic changes in *Monascus*, at transcriptional, translational, and metabolite levels. Such altered and complex intracellular metabolism appears to be the major determinant of pigment yield and diversity.

As a typical secondary metabolic pathway, MP biosynthesis uses polyketone synthesis (PKS) and fatty acid synthesis (FAS) to generate the core carbon skeleton, and then uses postsynthetic modifications to construct the various MP structures. The genes required for MP synthesis are linearly arranged in a gene cluster on chromosome V of *Monascus* spp. (Fig. 4a). Studies focusing on this pigment cluster have revealed the genetic mechanisms of *Monascus* pigment biosynthesis (26, 43–47). The dynamic expression of this gene cluster allows *Monascus* versatile pigment regulation, contributing to pigment diversity. In this study, using correlation analyses, both MOP and MYP synthesis were related to the expression of *MpigE*, *-N*, *-M*, *-D*, and *-K*, and structural genes for MP synthesis. MRP synthesis was significantly affected by the regulatory gene *MpigI*, which was highly coexpressed with the other two regulatory genes, *MpigP* and *-L*, and the dehydrogenase genes *MpigH*, *-F*, *-G*, and *-C*. This demonstrated that gene cluster expression was controlled by a sophisticated regulatory process. However, associations were at medium or low levels (*r *< 0.7). For example, cluster gene expression using AmS and AmCl was not higher than YEP, although MP production was far higher, suggesting gene cluster expression was not the sole determinant of MP production.

Additionally, given the low specificity of 4-*O*-acyltransferase (encoded by *MpigD*), which transfers the fatty acyl group to produce a mature MP molecular skeleton, and only a -C_2_H_4_- group difference between the fatty acyl chains of these three pairs of MPs, correlation analysis cannot completely distinguish the specific synthetic features of R_1_*/*R_2_, O_1_*/*O_2_, and Y_1_*/*Y_2_.

MOPs and MYPs are converted from a shared precursor by redox reaction (3). Correlation analyses of pigment production indicated that MOPs and MYPs interconverted directly, since their correlation coefficients were greater than 0.8, regardless of sampling times and nitrogen source forms. Previous studies reported that enzyme-mediated redox transformations were reversible, i.e., the *in vitro* interconversion of MOPs and MYPs (3). Such interconversion via hydrogenation/dehydrogenation reactions promoted MOP and MYP coproduction, but other factors, such as precursor availability and reduction equivalents (NAD[P]H), complicated interconversion regulation and hindered the practical directional engineering of pigment diversity. In addition, *MpigF* and *MpigH* (encoding the dehydrogenases which produce MYPs and MOPs [3]) were highly coexpressed with *MpigI*, *-L*, and *-P* regulatory genes, suggesting a potential regulatory interconversion mechanism between MOPs and MYPs.

MRPs are biosynthesized by the nonenzymatic amination of MOPs (3), but correlation analyses of pigment interconversion suggested a weak connection between MRPs and MOPs/MYPs. This was not unexpected, as conversion to MRPs depended on the supply of synthetic precursors, including MOPs, amines, and amino acids. Furthermore, correlation analyses showed a distant relationship between MRP biosynthesis and gene cluster expression. Clearly, the main limiting factors to MRP production were outside the gene cluster. Using metabolomics, metabolites, including amino acids and their derivatives, had a strong influence on MRP biosynthesis, supporting the viewpoint that MRPs are produced by amination. Unlike MRPs, the metabolites associated with MYP and MOP biosynthesis were involved with nucleotides, suggesting the biosynthetic regulation of MRPs and MYPs/MOPs is significantly different at the metabolomic level.

WGCNA highlighted associations between pigment diversity and functional modules. Using this approach, genes with similar expression patterns were clustered and associations between gene modules and physiological characteristics were revealed. KEGG pathway enrichment analyses highlighted the biological importance of these modules. WGCNA-based correlation analyses between transcriptome and phenotypic traits characterized specific regulation mechanisms for pigment diversity. In addition, each pair of pigment homologs, i.e., R_1_/R_2_, O_1_/O_2_, and Y_1/_Y_2_ had similar metabolic regulatory patterns. Nevertheless, all pigments appeared to be subjected to a comprehensive regulatory process from the pigment biosynthetic gene cluster, central carbon metabolism, and other potential factors in genomic pathways. Thus, WGCNA clearly showed a number of primary metabolic pathways were closely involved with MRP production. This was verified by our metabolomic analyses; MRPs were closely related to amino acids and intermediates from central carbon metabolism, whereas MOPs and MYPs were putatively regulated by nucleotides, but independent of central carbon metabolism. The latter finding was also validated by WGCNA.

In summary, our combined analytical multiomics strategy provided a technical platform and new perspectives for unveiling the metabolic shunting mechanism of MP biosynthesis. We improved our understanding of intracellular metabolic alterations during MP differentiation. These findings will be important for future metabolic *Monascus* engineering and MP optimization.

## MATERIALS AND METHODS

### Microorganisms and media.

*Monascus purpureus* M7 spores were deposited in our laboratory and stored at −80°C in 20% glycerol aqueous solution. The solid agar medium potato dextrose agar (PDA) (https://core.ac.uk/display/23854048) was used for plate analyses. Seed medium composition was rice flour (30 g/liter), NaNO_3_ (3 g/liter), KH_2_PO_4_ (2.5 g/liter), MgSO_4_·7H_2_O (1.0 g/liter) (pH 5.8 to 6.0). Liquid fermentation medium for *Monascus* culture was composed of fixed and variable components. Fixed components were glucose (50 g/liter), KH_2_PO_4_ (4.0 g/liter), MgSO_4_·7H_2_O (0.5 g/liter), MnSO_4_·H_2_O (0.03 g/liter), ZnSO_4_·7H_2_O (0.01 g/liter), and FeSO_4_·7H_2_O (0.01 g/liter). Nitrogen sources comprised the variable component and came from one of the following nitrogen chemical forms: (NH_4_)_2_SO_4_ (5.0 g/liter) (AmS), NH_4_Cl (5.0 g/liter) (AmCl), urea (5.0 g/liter) (Ur), glycine (5.0 g/liter) (Gly), glutamate (5.0 g/liter) (Glut), sodium nitrate (5.0 g/liter) (SoN), ammonium nitrate (5.0 g/liter) (AmN), peptone (20 g/liter) (Pep), and yeast extract powder (20 g/liter) (YEP). The initial pH of the fermentation medium was adjusted to 5.8 with 1M NaOH and HCl. All media were sterilized at 121°C for 20 min.

*Monascus* fermentation was performed in 250-ml Erlenmeyer flasks using a 10% (vol/vol) inoculation volume and a 40 ml working volume at 30°C for 4 days in a shaking incubator at 180 rpm (ZQWY-200N, Zhichu Instrument Co., Ltd., Shanghai, China). Fermentation studies were repeated at least three times.

### Analytical methods for phenotypic characteristics.

Fermentation broth (10 ml) was centrifuged at 2,000 × *g* for 15 min (TDZ5-WS, Xiangyi Centrifuge Instrument Co., Ltd., Shanghai, China) to generate a supernatant for glucose determination and a mycelial pellet for the extraction of six major alcohol-soluble pigments (monascin, ankaflavin, rubropunctatin, monascorubrin, rubropunctamine, and monascorubramine, designated as Y_1_, Y_2_, O_1_, O_2_, R_1_, and R_2_, respectively).

Mycelial pellets were soaked in aqueous ethanol (5 ml, 70%, vol/vol) at 60°C for 1 h, then ultrasonicated for 30 min in an ultrasonic cleaning bath (KQ8200B, Kunshan Ultrasonic Instrument Co., Ltd., Shanghai, China) and centrifuged at 2,000 × *g* for 15 min. The supernatant was filtered through a 0.22-μm filter and analyzed by high-performance liquid chromatography (HPLC) (Agilent 1200, Agilent Technologies Inc., Palo Alto, CA, USA), equipped with a ZORBAX SB-C_18_ column (250 × 4.6 mm, 5 μm, Agilent Technologies) and a Diode-Array Detection detector at 410 nm. The isocratic mobile phase was set at 1.0 ml/min and composed of water (containing 0.1% formic acid, vol/vol) and acetonitrile at a 35:65 ratio.

Residual glucose (RG) in the medium was determined by HPLC (Thermo U-3000, Thermo Fisher Scientific, Waltham, MA, USA) on an Aminex HPX-87H column (Bio-Rad Laboratories Inc., 300 × 7.8 mm, 9 μm, Hercules, CA, USA) and a refractive index (RI) detector. The mobile phase was 5 mM aqueous sulfuric acid set at a 0.6 ml/min flow rate. The column was maintained at 60°C. Mycelia were dried to determine the biomass as dry cell weight (DCW).

If not specified otherwise, Student's *t* test for independent samples was used for statistical analysis to compare the difference between the means of two groups using R 4.0.

### Metabolomic analysis.

Mycelia of 5 ml of fermentation broth were collected and washed in precooled phosphate-buffered saline (PBS) (KH_2_PO_4_ [0.24 g/liter], Na_2_HPO_4_ [1.44 g/liter], NaCl [8 g/liter], KCl [0.2 g/liter], dissolved in deionized water, pH 7.2). Wet mycelia (1.0 g) were treated with methanol/acetonitrile/H_2_O (1.5 ml; 40:40:20 vol/vol/vol) at −20°C for 60 min to extract intracellular metabolites. After freezing and thawing in liquid nitrogen at least five times, extracts were centrifuged at 5,000 × *g* at 4°C for 10 min and supernatants retained to determine metabolite profiles using ultra-HPLC (Waters Co., Waltham, MA, USA) coupled to a Q Exactive Hybrid Quadrupole-Orbitrap mass spectrometer (Thermo Fisher), as described previously (48), with negative ion mode. Each metabolomic study was repeated four times. Xcalibur 4.0 software (Thermo Fisher) was used for data acquisition and processing. Metabolite identification was conducted using high-resolution mass and retention-time matching to authentic standards. Metabolite abundance was normalized to wet mycelium weight.

### Transcriptome sequencing.

Mycelia of 10 ml of fermentation broth at 48 h were harvested for transcriptomic sequencing on the Illumina Hiseq platform (Illumina, Inc., San Diego, CA, USA). RNA isolation, library preparation, sequencing, data filtering, and quality control were performed as described previously (30). The reference genome was *M. purpureus* strain YY-1 and gene annotation files were downloaded from the National Center for Biotechnology Information (https://www.ncbi.nlm.nih.gov/genome/17783) (30). The expected number of fragments per kilobase of transcript sequence per million base pairs sequenced (FPKM) was calculated based on length and read counts mapped to each gene (49). Each transcriptomic study was repeated three times.

### Correlation analyses.

Relationships between phenotypic characteristics (production of the major six MPs, RG, and DCW), intracellular metabolite abundance, and gene expression levels were measured by calculating the Pearson correlation coefficient *r*, using SPSS. A *P* value < 0.05 was used in all cases.

### Construction of the WGCNA network.

WGCNA was built from transcriptome data sets using FPKMs, following the online R 3.5.1 tutorial (https://horvath.genetics.ucla.edu/html/CoexpressionNetwork/Rpackages/WGCNA/Tutorials/) (36). Briefly, a similarity matrix was constructed by calculating Pearson correlation coefficients between sample gene expression profiles. Then, the matrix was transformed into an adjacency matrix (A) raised to a β exponent (soft threshold) based on free-scale topology criterion. The topological overlap matrix (TOM) was used to define modules based on dissimilarity (1-TOM). Genes with highly similar correlation relationships were grouped into the same modules through hierarchical clustering based on results from TOM. Each gene module was assigned a standard default RGB color, and genes not grouped into modules were assigned to the Gray module. Module-trait associations were estimated using correlations between the module eigengene and physiological characteristics, including DCW, RG, and MPs, permitting the identification of modules highly correlated with the traits of interest. Genes with significant module-trait associations (coefficient > 0.60 and *P* value < 0.001) within modules were characterized using KEGG pathway analyses.

### Data availability.

The data sets used and/or analyzed here are available from the corresponding author upon reasonable request.
