# Supplementary material for: Integrative Metabolomic and Transcriptomic Analyses Uncover Metabolic Alterations and Pigment Diversity in Monascus in Response to Different Nitrogen Sources
Source: mSystems. 2021 Sep 7;6(5):e00807-21. doi: 10.1128/mSystems.00807-21 (PMC8547423; doi:10.1128/mSystems.00807-21)
Supplement: FIG S1 [file msystems.00807-21-sf001.pdf]

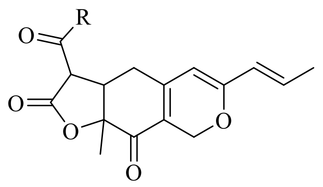

$Y_1$ Monascin $R=C_5H_{11}$
$Y_2$ Ankaflavin $R=C_7H_{15}$

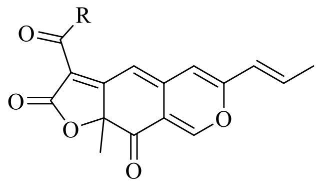

$O_1$ Rubropunctatin $R=C_5H_{11}$
$O_2$ Monascorubrin $R=C_7H_{15}$

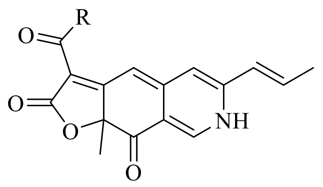

$R_1$ Rubropunctamine $R=C_5H_{11}$
$R_2$ Monascorubramine $R=C_7H_{15}$
